# Masked pressure lesion on the tip of the toe, a diagnostic challenge: Case report

**DOI:** 10.1007/s00508-026-02732-w

**Published:** 2026-03-17

**Authors:** Regina Fink-Puches, Linda Irmler, Maximilian Zarfl

**Affiliations:** Dermatology, Medical University of Graz, Auenbruggerplatz 8, 8036 Graz, Austria

**Keywords:** Malignant melanoma, Amelanotic melanoma, Fibromatous lesion, Acral melanoma, Oncodermatology

## Abstract

Amelanotic acral melanoma is an uncommon melanoma subtype often associated with delayed diagnosis due to its nonpigmented and clinically inconspicuous presentation. We report the case of a 61-year-old man presenting with a painful erythematous nodule on the plantar aspect of the second toe persisting for 1 year and initially presumed benign. Histopathological evaluation confirmed acral amelanotic melanoma (pT3a) with micrometastatic sentinel lymph node involvement. The patient underwent partial toe amputation followed by adjuvant immunotherapy with nivolumab. This case underscores the importance of early biopsy of persistent acral lesions to reduce diagnostic delay and improve outcomes.

## Introduction

A 61-year-old patient presented at the dermatology department of the University Hospital Graz with a fibromatous lesion on the plantar side of the second toe of his left foot, present for about 1 year. It appeared after a foot massage. Over time, a round thickening developed with increasing pain and walking discomfort. Due to persistent symptoms, he was referred for further evaluation.

**Fig. 1:**
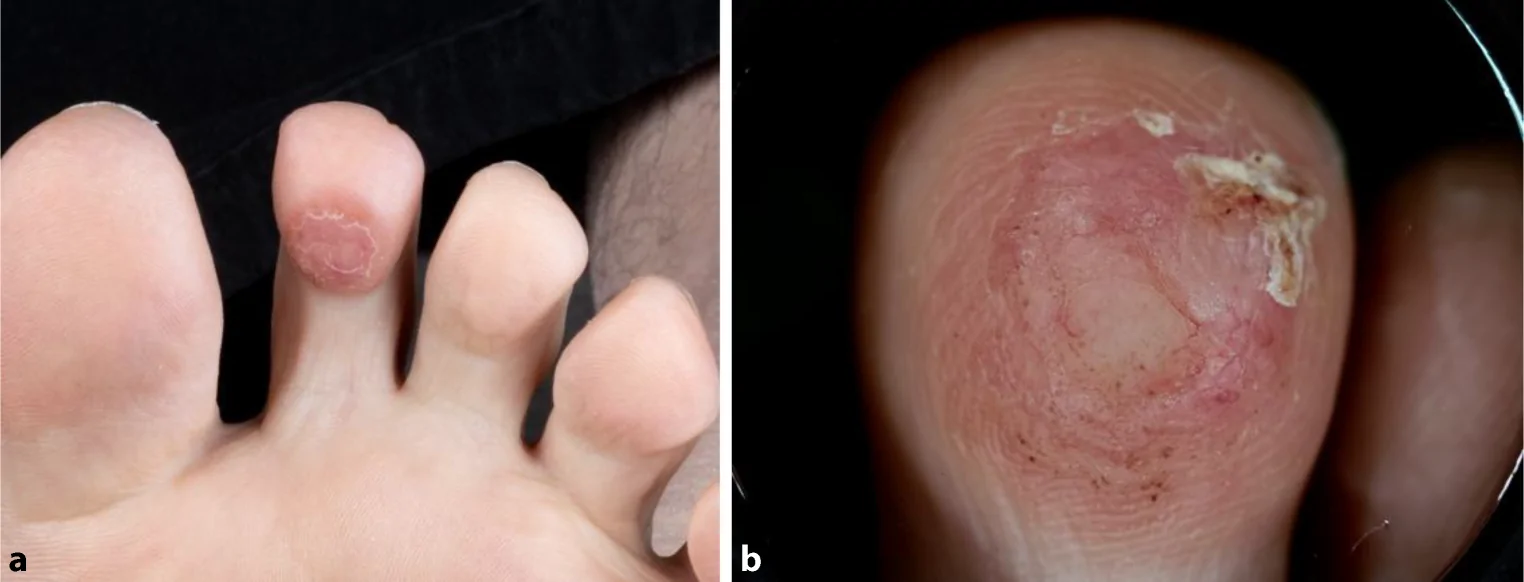
Diagnosis: acral amelanotic melanoma. **a** Native clinical photograph showing a fibromatous lesion on the plantar surface of the second toe. **b** Dermoscopic close-up image of the lesion obtained with a professional dermatoscope.

A soft, slightly scaly, 1 cm erythematous nodule with fibromatous, sharply defined appearance was found on the plantar side of the distal phalanx (see Fig. 1). Magnetic resonance imaging (MRI) showed dermal thickening with minimal contrast uptake on the plantar side of the second ray, suggesting a tumorous lesion. Photographic documentation was performed, and a punch biopsy taken for histopathological examination due to suspected fibroma or vascular tumor.

## Clinical course

Following histopathological diagnosis of malignant melanoma in January 2025 (punch biopsy; tumor thickness 2.3 mm; left second toe; pT3a), staging revealed no distant metastases.

In February, a sentinel lymph node was removed from the left inguinal region during surgical treatment. Histology showed tumor positivity with cells laterally (20 μm) and medially (30 μm), corresponding to pN1a(i+)(sn).

Adjuvant systemic immunotherapy with nivolumab was started. Subsequently, partial amputation of the affected toe was performed (acral melanoma; tumor thickness 3.5 mm; pT3a i; resection margins sano; left second toe).

## Discussion

In this case, the patient assumed that the fibromatous skin changes on his foot, which initially developed according to his medical history after a foot massage, which is why he subjectively classified the skin change as harmless and only consulted a dermatologist after it persisted for a long time. The general practitioner (GP) was also contacted and assessed the lesion also as harmless.

Malignant skin changes are often initially diagnosed as verrucae, mycotic diseases, keratoacanthomas or blisters. For example, a 42-year-old Chinese female patient was misdiagnosed by 5 dermatologists with a plantar verruca on the left sole of her foot. Finally, a diagnosis of amelanotic malignant melanoma was made after a biopsy was performed 6 months later due to a secondary swollen lymph node [1].

Clinically, amelanotic malignant melanoma can present in many different ways. In our case, a 2 cm erythematous, sharply and regularly defined, slightly scaly skin lesion with a fibromatous appearance was found on the distal phalanx of the second toe.

In the context of medical history and early detection, the fair and sun-sensitive phenotype (characterized by red hair, fair skin and numerous freckles) should be considered a potential risk factor for amelanotic melanoma. According to Misiaq et al., this phenotype can be associated with certain *MC1R* gene variants. The MC1R R/R and r/r genotypes were found significantly more frequently in patients with amelanotic melanoma than in those with pigmented melanoma [2]

Despite the fact that acral amelanotic melanomas predominantly occur on the sole of the foot (53.8%) and the heel (25%) compared to the hand (21.2%), histological examinations in a further study were performed on the sole of the foot in only 7.1% of cases and on the heel in 0% of cases, while a biopsy was performed on the hand in 50% of cases. This diagnostic reluctance in the lower extremities is also a significant factor in delayed diagnosis and thus a potential worsening of the prognosis [3].

Amelanotic melanomas, and in particular subtypes such as desmoplastic melanoma, also pose a clinical challenge in dermoscopy as they are often pigment-free or have an appearance and can therefore easily be confused with benign lesions. In our patient, due to the seemingly clear clinical picture, dermoscopy was performed late (only after histological diagnosis), revealing even minimal pigment content. It is important to pay special attention to the lesion even if the pigment content is minimal [4].

Dermoscopy revealed microscopic features, in particular atypical vascular structures of varying size and shape, punctate vessels, and a central whitish-bluish-pink haze [5].

Dermoscopy revealed the hemorrhagic crust of the punch biopsy in the upper area of the lesion in our patient. Atypical branched vessels of varying thickness are noticeable in the center. In the lower edge area, discreetly pigmented globules of varying sizes can be seen, which are predominantly located on the grooves.

In summary, amelanotic malignant melanomas are often detected late and therefore require a high degree of clinical vigilance. Dermoscopy plays a crucial role in this context, as it can reveal subtle microscopic features that are not identifiable on routine clinical examination. Early histological clarification of even inconspicuous-looking lesions is essential to improve the prognosis.
